# Clotting Without a Clue: A Case Report of Acute Pulmonary Thromboembolism as the Initial Presentation of Focal Segmental Glomerulosclerosis (FSGS)-Associated Nephrotic Syndrome Without Deep Vein Thrombosis

**DOI:** 10.7759/cureus.88336

**Published:** 2025-07-19

**Authors:** Elangovan Raman, Manimaran Rajendran, Magesh Rajasekaran, Sanjay Shankar R, Sorabh Sharma, Diksha Indra Krishna Prasad

**Affiliations:** 1 Internal Medicine, Madras Medical College and Rajiv Gandhi Government General Hospital, Chennai, IND; 2 Emergency Medicine, Madras Medical College, Chennai, IND; 3 General Medicine, Saveetha Institute of Medical and Technical Sciences, Chennai, IND; 4 Internal Medicine, University of Arizona College of Medicine - Tucson, Tucson, USA; 5 Paediatrics, Jawaharlal Institute of Postgraduate Medical Education and Research, Pondicherry, IND

**Keywords:** focal segmental glomerulosclerosis (fsgs), life threatening, pulmonary thrombo-embolism without dvt, renal biopsy in nephrotic syndrome, young male

## Abstract

We report the case of a 23-year-old male from rural South India who presented to the emergency department with sudden-onset dyspnea, exacerbated by exertion. He had a recent diagnosis of chronic kidney disease (CKD) and was on irregular outpatient treatment. A CT pulmonary angiogram revealed bilateral pulmonary artery filling defects, consistent with pulmonary embolism (PE). Laboratory workup showed significant proteinuria, dyslipidemia, hypoalbuminemia, and elevated hematocrit. A renal biopsy performed after stabilization confirmed focal segmental glomerulosclerosis (FSGS) with the perihilar variant. The patient was managed with anticoagulation, high-dose steroids, and supportive care. This case highlights the potential for thromboembolic events, such as PE, to serve as the initial manifestation of nephrotic syndrome (NS), even in the absence of deep vein thrombosis (DVT), emphasizing the need for early suspicion and prompt evaluation in young patients with unexplained respiratory symptoms.

## Introduction

Nephrotic syndrome (NS) is characterized by the presence of heavy proteinuria (typically >3.5 g/24 hours or protein-to-creatinine ratio >300-350 mg/mmol), hypoalbuminemia (<30 g/L), peripheral edema, and often hyperlipidemia [1]. The majority of NS cases are classified as idiopathic or primary, with the most common histopathological subtypes being focal segmental glomerulosclerosis (FSGS) and membranous nephropathy [2]. NS has an incidence of approximately three cases per 100,000 adults per year. It remains a rare manifestation of kidney disease compared to microalbuminuria caused by diabetes mellitus and systemic hypertension [3].

NS is considered a prothrombotic condition. This is believed to result from urinary loss of natural inhibitors of coagulation, such as antithrombin III and free protein S. Therefore, it should be considered in any patient presenting with an unprovoked embolic event [4]. Increased platelet aggregation and decreased levels of anticoagulants such as antithrombin III are also believed to contribute to a heightened risk of venous thrombosis [5].

Here, we present the case of a young man with pulmonary thrombus as the presenting feature of NS, without evidence of deep vein thrombosis (DVT).

## Case presentation

A 23-year-old man was admitted to the emergency department with complaints of shortness of breath, which was exacerbated on exertion and relieved with rest. There were no complaints of chest pain, palpitations, or diaphoresis. These symptoms developed two months after his initial diagnosis of renal failure (chronic kidney disease (CKD)), during which the patient had presented with facial puffiness, peripheral edema, and abdominal distention. He was prescribed diuretics and referred for further evaluation; however, in his own words, he was not compliant. He had no other medical history and reported consuming alcohol for the past six years.

On physical examination, he was tachypneic with bilateral pitting pedal edema. The remainder of the physical examination was unremarkable. His pulse was 128/min, respiratory rate was 24 breaths/min, and oxygen saturation was 85% on room air, which improved to 98% with a face mask O_2_ of 10 L/min. ECG showed sinus tachycardia with an S1Q3T3 pattern. Initial laboratory investigations revealed a urea level of 45 mg/dL, creatinine of 1.4 mg/dL, hemoglobin of 18.9 g/dL, and hematocrit of 54.7%. Prothrombin time (PT) was 13.74 seconds with an international normalized ratio (INR) of 1.07; activated partial thromboplastin time (APTT) was 21.5 seconds, indicating no coagulation abnormality. Erythropoietin was 5.98 mIU/mL. D-dimer value was elevated at 3.16 µg/mL. Serum albumin was 2.3 g/dL, and total protein was 5.6 g/dL. All other blood investigations were within normal limits (Table 1).

**Table 1:** Blood investigations ALT: alanine transaminase; AST: aspartate aminotransferase; APTT: activated partial thromboplastin time; INR: international normalized ratio; HDL-C: high-density lipoprotein cholesterol; LDL-C: low-density lipoprotein cholesterol; PT: prothrombin time; SGOT: serum glutamic-oxaloacetic transaminase; SGPT: serum glutamic-pyruvic transaminase; WBC: white blood cell; VLDL-C: very low-density lipoprotein cholesterol

Test	Result	Reference Range
Hemoglobin	18.9 g/dL	13.3–16.2 g/dL
Hematocrit	54.70%	38.8–46.4%
WBC	12,400 /μL	3,500–9,060 /μL
Platelet	185,000 /μL	165,000–415,000 /μL
Erythropoietin	5.98 mIU/mL	4.30–29 mIU/mL
PT	13.74 seconds	12.7–15.4 seconds
INR	1.07	0.9–1.5
APTT	21.5 seconds	21–39.4 seconds
Urea	17 mg/dL	15–40 mg/dL
Creatinine	0.8 mg/dL	0.5–1.3 mg/dL
Total Bilirubin	0.4 mg/dL	0.3–1.3 mg/dL
Direct Bilirubin	0.1 mg/dL	0.1–0.4 mg/dL
AST (SGOT)	22 IU/L	12–38 IU/L
ALT (SGPT)	15 IU/L	7–41 IU/L
Total Cholesterol	344 mg/dL	0–200 mg/dL
Triglyceride	125 mg/dL	40–160 mg/dL
HDL-C	47 mg/dL	40–60 mg/dL
LDL-C	272 mg/dL	50–100 mg/dL
VLDL-C	25 mg/dL	2–30 mg/dL
D-dimer	3.16 µg/mL	< 0.5 µg/mL
Troponin	609 ng/L	22 ng/L
NT-proBNP	2718 pg/mL	125–163 pg/mL
LDH	543 IU/L	140–280 IU/L
HBsAg	Negative	
HCV–DNA PCR	Negative	
HIV	Negative	
JAK2 EPO Mutation	Negative	
Protein C/S Mutation	Negative	
Antithrombin III	Negative	
Factor V Leiden Mutation	Negative	
ANA (Antinuclear Antibody)	Negative	
Lupus Anticoagulant	Negative	
Anticardiolipin Assay	Negative	
Homocysteine	Negative	

Urinalysis revealed 3+ proteinuria on dipstick, with a urine protein-creatinine ratio (PCR) of 5.27, consistent with nephrotic-range proteinuria. Urine tested negative for ketones, hematuria, bilirubin, nitrites, urobilinogen, and glucose. The fasting lipid profile demonstrated marked hypercholesterolemia, with a total cholesterol level of 344 mg/dL, while other lipid parameters remained within normal limits.

Transthoracic echocardiography showed a D-shaped left ventricle, indicative of right ventricular pressure overload. There was notable dilatation of both the right atrium and right ventricle. Left ventricular systolic function was preserved with an ejection fraction of 60%. Additionally, features suggestive of severe pulmonary arterial hypertension were observed.

In view of his clinical presentation and laboratory findings, differential diagnoses included pulmonary embolism (PE), pneumonia, and acute coronary syndrome (ACS). A Wells score of 4.5 supported an intermediate to high probability of PE [6]. Empirical treatment was initiated with intravenous heparin (25,000 units at 6 mL/hour) and ceftriaxone 1 g twice daily.

Although pneumonia was initially suspected, the absence of fever, productive cough, and radiological infiltrates made this less likely. ACS was also considered in light of sinus tachycardia and elevated troponin levels; however, the electrocardiogram demonstrated an S1Q3T3 pattern - more consistent with acute right heart strain secondary to PE and lacked ischemic ST-T changes typically seen in myocardial infarction. NT-proBNP was significantly elevated, further supporting right ventricular strain as the etiology rather than left-sided cardiac dysfunction.

**Figure 1:**
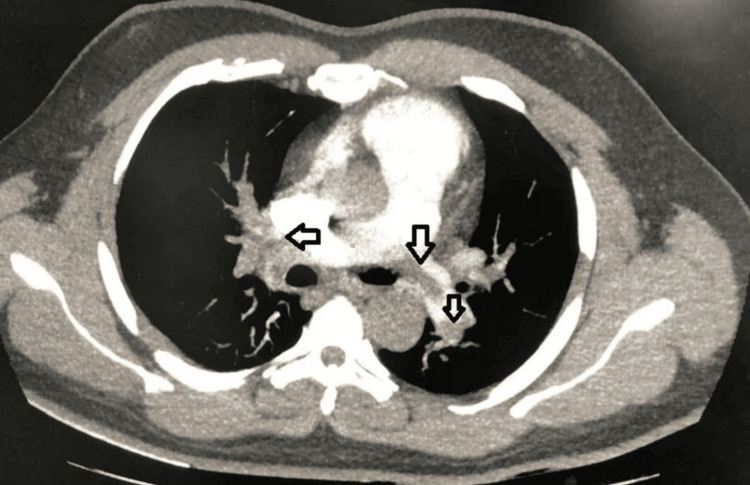
CTPA - A hypodense, non-enhancing filling defect is noted within the main pulmonary artery extending into the left pulmonary artery, resulting in near-complete occlusion (corresponding to the middle arrow) Additional hypodense intraluminal filling defects are identified in the distal branches of the right pulmonary artery and the distal left pulmonary artery, consistent with bilateral pulmonary embolism.

**Figure 2:**
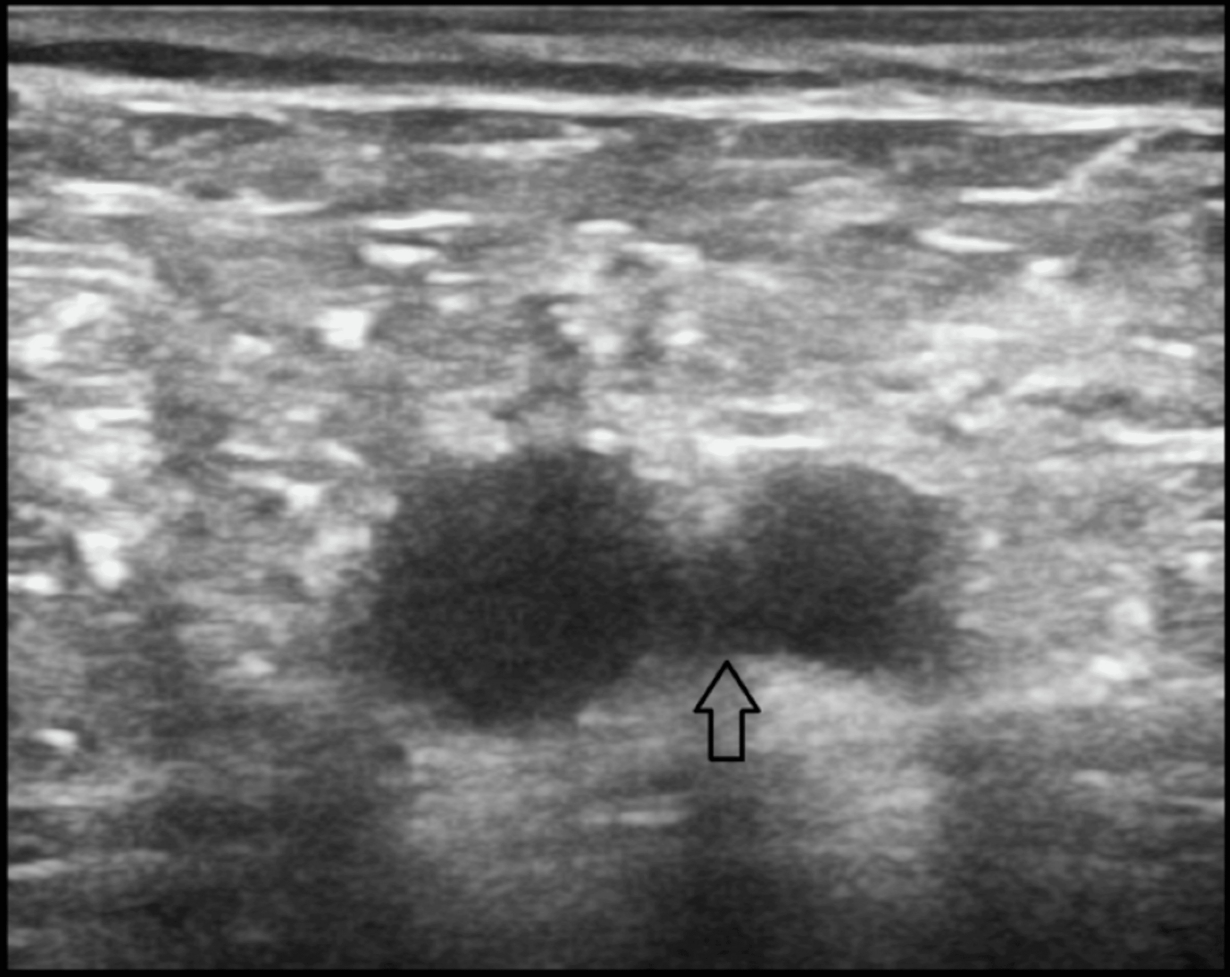
Lower limb venous Doppler studies revealed no thrombus - negative deep venous thrombosis

CT pulmonary angiography (CTPA) confirmed the diagnosis of acute PE, revealing non-enhancing hypodense filling defects in the distal right pulmonary artery and its segmental branches, as well as a near-complete occlusion in the left pulmonary artery (Figure 1). Bilateral lower limb venous Doppler studies ruled out DVT (Figure 2). Additionally, infectious disease workup, including HIV, HBsAg, and hepatitis C virus (HCV) serologies, was negative. These findings conclusively established pulmonary embolism in the context of an underlying hypercoagulable state, likely driven by nephrotic syndrome, as the primary diagnosis.

The nephrology team was consulted, and the patient was started on heparin, albumin, furosemide, and telmisartan. After two weeks, the patient was deemed fit for discharge and was sent home on the same medications, with heparin replaced by oral anticoagulant.

**Figure 3:**
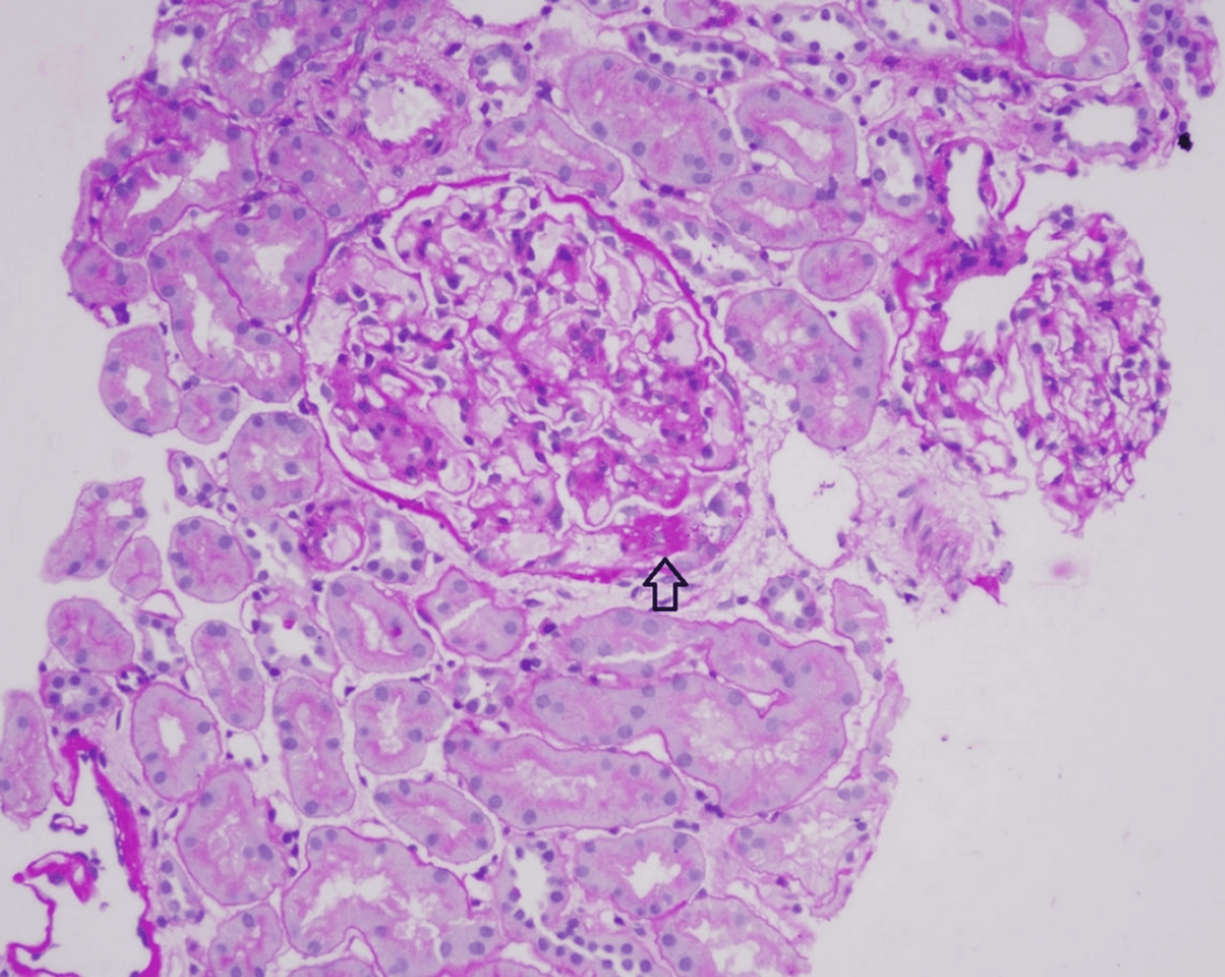
A renal biopsy showing segmental sclerosis of the capillary tuft with adhesions to Bowman’s capsule in the perihilar region in one glomerulus, consistent with nephrotic syndrome of the focal segmental glomerulosclerosis (FSGS) subtype

A renal biopsy was performed one month after discharge, following the temporary discontinuation of oral anticoagulation. Histopathological examination revealed segmental sclerosis of the glomerular capillary tuft with adhesion to Bowman’s capsule in the perihilar region of one glomerulus, consistent with the perihilar variant of focal segmental glomerulosclerosis (FSGS) (Figure 3). Based on these findings, the patient was initiated on high-dose corticosteroid therapy with prednisolone 60 mg once daily. Oral anticoagulation was reintroduced concurrently, and a structured follow-up plan was established with the nephrologist.

## Discussion

Patients with NS are at an increased risk for thromboembolic events, such as DVT, arterial thrombosis, cerebral venous thrombosis, and PE. This is attributed to a state of "hypercoagulability" resulting from a combination of proteinuria, hypercholesterolemia, and hypoalbuminemia [7]. NS is a well-known risk factor for arterial or venous thromboembolism, and patients with severe proteinuria have a 3.4-fold higher risk of venous thromboembolism [8]. Thrombosis may arise in NS due to the loss of proteins involved in the inhibition of systemic hemostasis, such as antithrombin III deficiency, along with increased synthesis of prothrombotic factors or local activation of the glomerular hemostatic system [9].

In a pooled analysis of 11 studies conducted by Leslom et al., the estimated prevalence of PE among individuals with NS was reported to be approximately 7.93% [10]. Among various thromboembolic complications observed in NS patients, PE accounted for 27% of cases, with renal vein thrombosis being the second most frequent, seen in 13% of affected individuals [11].

PE as the first presenting symptom of NS, in the absence of DVT, is an extremely rare occurrence - reported in less than 5% of cases - making this case an important contribution to the literature [12]. In our case, the unexpected finding was the rare occurrence of early-onset thromboembolism in the absence of prior deep vein or renal vein thrombosis. In our literature review, we found that adults have approximately a seven- to eightfold increased incidence of thrombosis compared with children [5]. High rates of urinary protein excretion are associated with an increased thrombotic state in patients with NS. A study by Li et al. showed that, out of 100 patients with membranous nephropathy, those with thrombus had higher proteinuria (7.98 g/dL) compared with those without thrombus burden (6.39 g/dL), and the odds ratio was 1.18 (95% CI: 1.02-1.35; p = 0.02) for developing VTE [13]. In a large cohort of 898 patients with biopsy-confirmed membranous nephropathy, Lionaki et al. observed that venous thromboembolism risk increased significantly as serum albumin declined, with a 3.9-fold rise below 2.8 g/dL and a 5.8-fold increase when levels dropped below 2.2 g/dL [14]. The interval following diagnosis appears to influence VTE occurrence significantly. In a retrospective analysis involving 298 NS patients, 9.85% were diagnosed with thromboembolism within six months of initial presentation [15]. According to findings by Kumar et al., approximately one-fifth of patients with membranous nephropathy experienced thromboembolic events, with 64% presenting with VTE at diagnosis and the remainder developing it within the first six months [16]. Lionaki et al. found that, in MN, the median time to VTE occurrence was 3.8 months [14].

Despite the lack of conclusive evidence, corticosteroids remain the mainstay of treatment for NS. Immunosuppressive drugs may be considered for patients who are resistant to steroids or other medical management under the guidance of a nephrologist [17]. For our patient, anticoagulation was discontinued, followed by a renal biopsy. Prior to discharge, he was started on high-dose corticosteroids.

In our patient, the young age of 23, high proteinuria as evidenced by the urine protein-creatinine ratio, hypoalbuminemia, and the recent diagnosis of NS placed him in a high-risk cohort for developing VTE. Prevention of thromboembolism in patients with NS using anticoagulants has been a subject of debate, largely due to their adverse effect profile. This is of concern, as the majority of NS patients do not develop thromboembolism and may receive prophylaxis unnecessarily. The potential of statin therapy to reduce NS-associated thromboembolism has recently been explored in a study by Resh et al. [18]. A better alternative strategy is to identify high-risk individuals based on established risk factors and target them for prophylactic therapy. This approach would reduce the number needed to treat and avoid exposing low-risk patients to unnecessary therapeutic risks.

## Conclusions

This study highlights the critical hypercoagulability in NS, where life-threatening events such as PE and CVT may be the initial presentation, even without prior symptoms. Physicians must remain vigilant for prompt diagnosis and treatment. Although massive proteinuria and age are known risk factors for thromboembolism in NS, alongside hemostatic abnormalities, no reliable clinical biomarker currently exists. Future research should prioritize identifying predictive biomarkers for thromboembolic risk. Despite guideline recommendations, the role of prophylactic antiplatelets or anticoagulants remains uncertain; large meta-analyses could clarify these issues. Enhancing awareness among primary care providers and emphasizing long-term secondary prevention are essential next steps.
